# A Model of Subjective Cognition in Older Adults: Correspondence of cognitive complaints with wellbeing, mood symptoms, and cognitive performance

**DOI:** 10.1002/alz.091748

**Published:** 2025-01-03

**Authors:** Shana D. Stites, Carolyn Kuz, Camilla Van Geen, Bob Scavilla, Joesph Kable, Sarah Barber

**Affiliations:** ^1^ Department of Psychiatry, Perelman School of Medicine, University of Pennsylvania, Philadelphia, PA USA; ^2^ University of Pennsylvania, Philadelphia, PA USA; ^3^ Fourfront, LLC, Philadelphia, PA USA; ^4^ Georgia State University, Atlanta, GA USA

## Abstract

**Background:**

Beginning new Alzheimer’s disease (AD) treatments before AD symptoms are prominent would optimize the benefits of these disease slowing treatments. To accomplish this goal, clinicians must identify measures of early disease progression. As a step in doing this, we set out to characterize the relationships between cognitive complaints, wellbeing, cognitive performance, and metacognitive calibration in older adults in order to inform a model of cognition in typical older adults.

**Method:**

Sample of 344 adults aged 65+ completed an online task. *Cognitive complaints* were assessed with 7 domains on the Memory Function Questionnaire (MFQ). *Wellbeing* was measured with ratings of overall wellbeing, health, satisfaction with daily life, anxiety, depression, and Future Time Perspective (FTP) scale. *Cognitive performance* was captured through reaction times and accuracy on Forward and Backward Digit Span (DS) tasks and accuracy on a Dot Matrix Task (DMT). Participants provided confidence ratings before and after the DMT. *Metacognitive calibration* (MCC) was operationalized as the difference between trial accuracy reaction time and global post‐DMT confidence ratings. Results reported are statistically significant at P<0.05 unless otherwise stated.

**Results:**

Fewer cognitive complains, as indicated by higher reports on all 7 MFQ domains, were associated with better health, wellbeing, and satisfaction with daily life. Most domains (6 of 7) were associated with more expansive FTP, higher anxiety (5 of 7), lower depressive symptoms (7 of 7), higher post‐task confidence (4 of 7), and better MCC (4 of 7). For 89% of participants, confidence ratings either decreased or remained unchanged from before to after the DMT; in the full multivariable model, the group that had confidence increase after the DMT showed lower accuracy (logit = ‐3.71, 95%CI ‐5.80, ‐1.61). In the full multivariable model, the group with low MCC (more confident than quick) demonstrated worse DMT accuracy (logit = ‐1.91, 95%CI ‐3.37, ‐0.45) and worse backward DS performance (logit = 0.25, 95%CI ‐0.04, 0.54, p = 0.09) compared to the typical MCC group (confidence∼reaction time).

**Conclusion:**

Cognitive complaints, wellbeing, cognitive performance, post‐task appraisals of confidence in task accuracy, and the consistency between perceptions and performance (metacognition) are useful constructs for informing a model of subjective cognition in typical older adults.

